# Investigating the Variables Associated with Having a Functional Limitation Among Adults with Long COVID: A Cross-Sectional Database Study Using the United States Medical Expenditure Panel Survey

**DOI:** 10.3390/diseases14090339

**Published:** 2026-09-16

**Authors:** David R. Axon, Stephanie Fenwick

**Affiliations:** James L. Winkle College of Pharmacy, University of Cincinnati, 3255 Eden Ave., Cincinnati, OH 45267, USA; bachmesf@ucmail.uc.edu

**Keywords:** long COVID, functional limitations, Medical Expenditure Panel Survey

## Abstract

Background/Objectives: Functional limitations are one of many potential clinical consequences for people living with long COVID. However, medical, health, and demographic variables associated with functional limitations in this population have not been fully characterized. The objective of this study was to identify variables associated with having a functional limitation among United States (US) adults with long COVID in a nationally representative dataset. Methods: This was a cross-sectional analysis of US adults in the 2023 Medical Expenditure Panel Survey (MEPS). A multivariable logistic regression model was created to identify factors associated with reporting functional limitations versus no functional limitations in people with MEPS-defined long COVID. Results: In this study, 26% of adults with MEPS-defined long COVID reported functional limitations. Factors that were associated with functional limitations in people with long COVID included non-married status versus married (odds ratio [OR] = 1.8), having 2–4 chronic health conditions versus 0–1 conditions (OR = 2.8), being unemployed versus employed (OR = 3.5), having public health insurance versus uninsured (OR = 5.3), and reporting pain interference versus no pain (little pain interference, OR = 2.3; moderate interference, OR = 8.8; extreme/quite a bit of interference, OR = 16.9). No other associations were found. Conclusions: Functional limitations are common in people experiencing long COVID. Several factors were associated with functional limitations in this population of individuals with long COVID, although whether the functional limitation was caused by long COVID or another problem was unknown. Further research is needed to better understand factors associated with long COVID and to explore the scope and severity of functional limitations in long COVID.

## 1. Introduction

For many individuals, the health burden associated with COVID-19 lasts well beyond the course of the initial infection. Long COVID is a sequela of SARS-CoV-2 infection that can have lasting implications for patients, with symptoms lasting months and even years. Although reports of extended symptoms after COVID-19 infection were reported early in the pandemic, medical consensus on a formal definition and name for this condition took several years to develop. In 2024, the National Academies of Sciences, Engineering, and Medicine (NASEM) developed a multifaceted consensus definition, describing long COVID as a chronic condition resulting from SARS-CoV-2 infection present for at least 3 months and involving at least one organ system [1]. The variable nature of long COVID symptoms and severity makes it a challenge to diagnose and treat. Moreover, our understanding of this condition is evolving, and many aspects are still poorly elucidated.

One of the consequences of long COVID for individuals is its potential to lead to functional limitations. Functional limitations are defined by the National Center for Health Statistics (NCHS) as difficulty performing or inability to perform in six key areas of functioning: sight, hearing, mobility, communication, cognition, and self-care [2]. Functional limitations can have severe consequences for the people affected. Psychological distress in the form of anxiety and depression has been associated with physical function limitations [3]. Moreover, inability to perform self-care tasks such as bathing, dressing, and cooking activities (typically described as activities of daily living [ADLs]) is associated with poor quality of life, cognitive decline, and increased mortality [4,5,6]. As long COVID is a relatively new disease, our understanding of the risk factors, prevalence, and implications of functional limitations associated with it is still incomplete.

Functional limitation in people with long COVID is likely a widespread problem—a 2022 study by Banić et al. stated that nearly 80% of their study population reported functional limitations lasting more than four weeks after an initial COVID-19 infection [7]. Women and people with hypertension or malignancy had a higher risk of functional impairment than other groups [7]. In a 2023 report, the Centers for Disease Control and Prevention (CDC) stated that over 25% of United States (US) adults with long COVID reported significant limitations in activity [8]. Individuals hospitalized for COVID-19 may have even more severe long-term functional limitations. Beauchamp et al. assessed outcomes in adult patients previously hospitalized with COVID-19 infection. At 12 months post-infection, over 55% of patients reported mobility deficits and over 38% reported cognitive deficits that were new since the COVID diagnosis [9]. Similar to the findings of Banić et al., women and individuals with more comorbid conditions were more likely to experience these challenges than other groups [9].

Functional limitations caused by long COVID have the potential to lead to a cascade of negative consequences for patients. A 2025 study by Prazeres et al. examined the effects of long COVID among healthcare workers. The researchers reported that long COVID was associated with a significant reduction in quality of life and reported symptoms of extreme fatigue and cognitive dysfunction were negatively correlated with work performance [10]. In another 2026 cross-sectional survey study of people with long COVID, over half of respondents were classified as having low quality of life. In that study, the factors with the strongest association with poor quality of life were symptom burden and employment [11]. Furthermore, a 2023 study by Vélez-Santamaría et al. found that functional status in long COVID predicted quality of life in participants [12].

Several factors have consistently been associated with a risk of functional impairment in people with long COVID, including female sex, more than one chronic disease, obesity, and hospitalization for acute COVID-19 [7,13,14]. However, there has been limited exploration into social and demographic factors that increase or modulate the likelihood of functional limitation in long COVID. Although factors such as race, ethnicity, economic instability, and access to health care have been implicated in the risk for developing long COVID itself [15], whether these or other factors are also associated with functional limitations in long COVID has not been extensively explored. As such, further research is essential to develop a better understanding of patterns and associations in people with functional limitations associated with long COVID.

The Medical Expenditure Panel Survey (MEPS) is a series of large, nationally representative surveys conducted by the Agency for Healthcare Research and Quality [16]. The MEPS survey gathers demographic and healthcare information on adults in the US, including survey questions that serve as indicators for the presence of long COVID and functional limitations [16]. The MEPS dataset offers an opportunity to explore medical, general health, and demographic factors impacting the lives of US adults and, as such, offers an opportunity to explore correlations between long COVID and functional limitations. The objective of this study was to identify the variables associated with having a functional limitation among US adults with long COVID.

## 2. Materials and Methods

This study examined data from MEPS, which is a series of large, nationally representative surveys that gather demographic and healthcare information on adults in the US. It includes three main components: the Household Component (MEPS-HC), the Medical Provider Component (MEPS-MPC), and the Insurance Component (MEPS-IC). MEPS-HC collects information directly from household members, whereas MEPS-MPC and MEPS-IC obtain data from medical and insurance providers, respectively, to supplement household reports. When the appropriate weighting variable is applied, MEPS can produce national estimates for the noninstitutionalized US population. The survey is reviewed and approved annually by an Institutional Review Board. For this study, the 2023 full-year consolidated data file was used, which contains information on 1374 variables for the 2023 calendar year [16,17,18].

### 2.1. Study Design and Eligibility Criteria

This cross-sectional study used the 2023 MEPS data (described above). For study inclusion, individuals had to be US adults over the age of 18, be alive for the full calendar year, and meet criteria for long COVID as defined by MEPS. To determine if an individual in the MEPS dataset had long COVID, surveyors asked participants if they had ever been diagnosed with COVID-19. If an individual said yes, then they were asked a second question to determine if they had symptoms lasting ≥3 months that they did not have prior to having COVID-19. Individuals who said yes to this second item were defined by MEPS as having long COVID [17,18].

### 2.2. Independent Variables

The independent variables in this study included age (70+, 60–69, 50–59, 40–49, 18–39), sex (male, female), race (White, Black, Asian, Multiple/other), ethnicity (Hispanic, non-Hispanic), marital status (never married/divorced/separated/widowed, married), education (up to and including high school, beyond high school), number of conditions (5+, 2–4, 0–1; from the following list: hypertension, coronary heart disease, angina, myocardial infarction, other heart disease, stroke, emphysema, chronic bronchitis, high cholesterol, cancer, diabetes, joint pain, arthritis, and asthma), regular exercise (yes, no), smoking status (smoker, nonsmoker), employment status (unemployed, employed), income (poor/low, middle/high), health insurance (private, public, uninsured), pain interference (extreme/quite a bit, moderate, little, none), physical health status (fair/poor, good, very good, excellent), and mental health status (fair/poor, good, very good, excellent) [17,18].

### 2.3. Dependent Variable

The dependent variable assessed in this study was functional limitation status. This dichotomous variable (functional limitation versus no functional limitation) was determined from an MEPS item that asked participants if they had any type of physical limitation [17,18].

### 2.4. Data Analysis

Data were analyzed using SAS (v9.4, SAS Institute Inc., Cary, NC, USA) software and the associated survey procedures. Differences between groups for each variable were compared using a chi-square test. A multivariable logistic regression model was constructed to assess the association between each independent variable and functional limitation versus no functional limitation. Multicollinearity was assessed by examining the variance inflation factor and was found not to be present. A significance level of alpha < 0.05 was selected a priori. Cluster and strata variables were used to maintain the integrity of the complex survey data. A weighting variable was used to calculate national estimates. The c-statistic indicated the discriminatory ability of the model. This study was approved by the University of Cincinnati Institutional Review Board (Protocol Number #2026-0538, 18 May 2026).

## 3. Results

**Figure 1:**
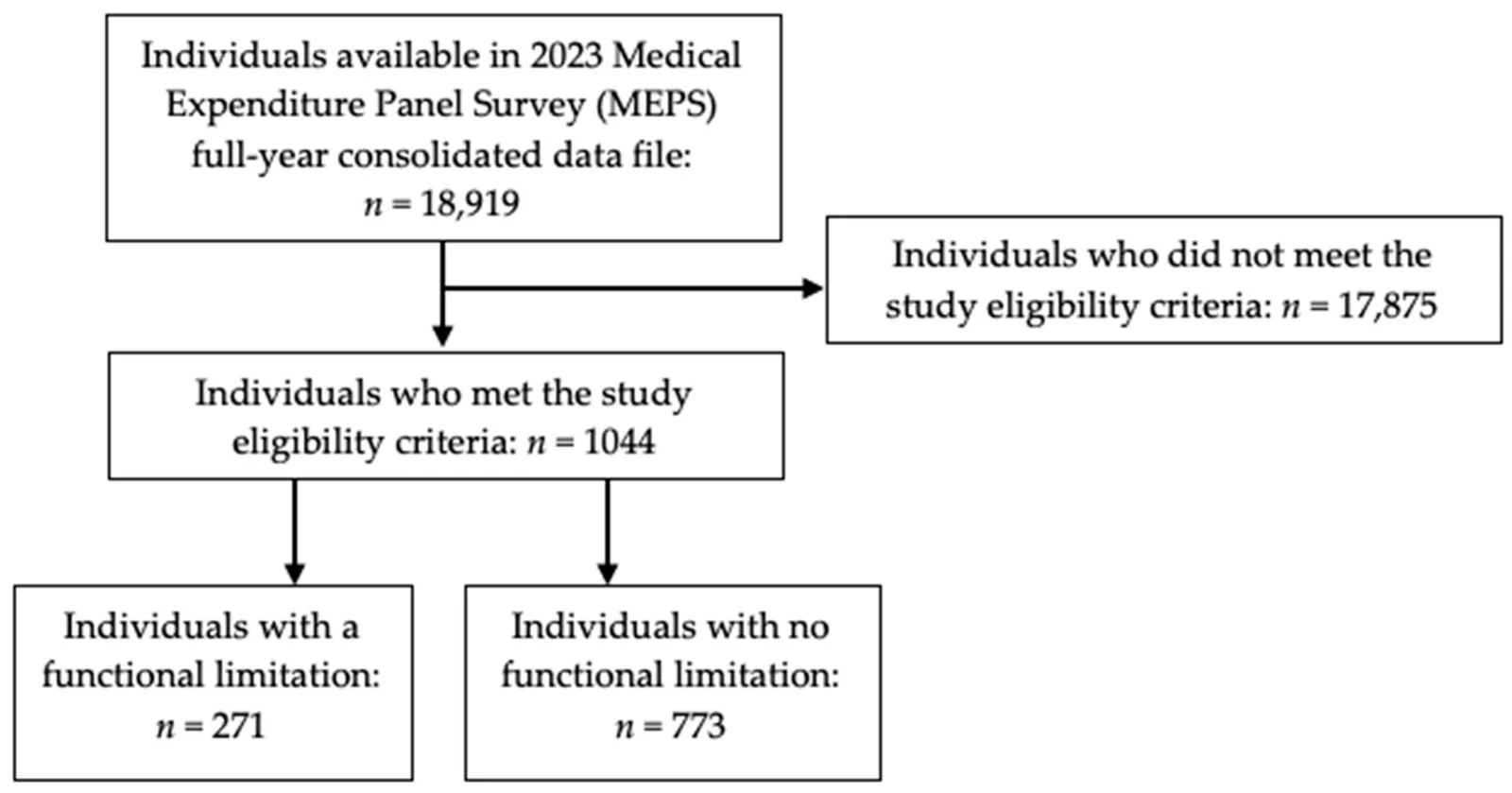
Study eligibility flowchart.

A total of 1044 individuals (weighted *n* = 27,120,935) with long COVID met the eligibility criteria of the study and were included in the analysis. Of these, 26% (*n* = 271) reported having a functional limitation (weighted *n* = 5,845,304) and 74% (*n* = 773; weighted *n* = 21,275,631) reported not having a functional limitation. See Figure 1.

### 3.1. Descriptive Data

In the study sample, the most common age group was 18–39-year-olds (31.7%), followed by 50–59-year-olds (21.9%), 60–69-year-olds (18.1%), 40–49-year-olds (16.3%), and those aged 70+ years (12.0%). The majority were female (57.4%), white (82.7%), non-Hispanic (82.0%), married (54.7%), educated beyond high school (59.0%), non-smokers (88.7%), employed (65.3%), had middle-to-high income (72.5%), and had private insurance (67.1%). Approximately half did not exercise regularly (50.9%). The most common number of conditions was 2–4 conditions (45.5%), followed by 0–1 conditions (40.4%), while the most common pain category was no pain (46.0%), followed by little pain (28.2%). Physical health was good, very good, or excellent for most individuals (78.9%), as was mental health (83.3%). Significant differences were observed between groups for all variables except sex, ethnicity, education status, and smoking status. See Table 1.

### 3.2. Multivariable Logistic Regression Model Results

In the multivariable logistic regression model, several variables were associated with increased odds of having a functional limitation among US adults with long COVID. These included marital status of never married/divorced/separated/widowed versus married; number of health conditions 2–4 versus 0–1; unemployed status versus employed; public health insurance versus uninsured; extreme/quite a bit pain interference versus none; moderate pain interference versus none; and little pain interference versus none. There was no association between the remaining variables and having a functional limitation in this analysis. See Table 2.

## 4. Discussion

In this investigation, 26% of surveyed individuals with MEPS-defined long COVID reported functional limitations. Several variables were associated with functional limitations: marital status, number of health conditions, employment status, health insurance status, and pain inference. No other variables had an association with having a functional limitation in this sample of US adults with long COVID. Although many of the factors identified in this study have been explored in relation to long COVID, understanding how these factors are associated specifically with functional limitations in long COVID allows for a more nuanced examination of this condition. One caution in interpreting these data is that the cause of the functional limitation is not captured by MEPS and may be unrelated to long COVID.

The presence of multiple chronic health conditions is commonly correlated with functional limitations in long COVID [7,14]. This was consistent with the findings of the present study, with people reporting 2–4 health conditions having higher odds of functional impairment compared with individuals with 0–1 conditions (OR = 2.8 [95% CI = 1.4, 5.6]). Interestingly, the odds of functional impairment for individuals with 5 or more conditions were not significantly increased compared with those with 0 or 1 condition (OR = 1.6 [95% CI = 0.7, 3.5]). This represents a non-monotonic pattern and may have been due to the relatively smaller number of individuals with 5 or more conditions in the study population (weighted *n* = 1,587,313 in the functional limitation group and weighted *n* = 2,252,475 in the no functional limitation group).

In examining demographic factors, in this study, people who were not married (never married/divorced/separated/widowed) had higher odds of functional limitations than those who were married (OR = 1.8 [95% CI = 1.2, 2.8]). Although marital status has not been reported specifically as an association with functional limitations in long COVID, there is some evidence that depressive symptoms and memory impairment are more common in people without spouses than those living with spouses after COVID [19]. Furthermore, Jace et al. report that mental health problems are more common in unmarried individuals versus married individuals after COVID-19 [20]. It is plausible that the mental health advantages experienced by married individuals could be protective for or reduce the perception of functional limitations, but further research is needed.

Participants who reported being unemployed had higher odds of functional limitations than employed respondents (OR = 3.5 [95% CI = 2.0, 6.4]). This follows what has been reported elsewhere regarding long COVID in general. Perlis et al. examined employment status in individuals with and without long COVID, determining that individuals with long COVID were more likely to be unemployed or not working full-time than individuals without long COVID [21]. More importantly, the researchers reported that the presence of any cognitive symptom, which could fall under the scope of a functional limitation according to the NCHS definition, was associated with a lower likelihood of full-time employment [2,21]. Furthermore, this may be a situation of reverse causality, where the unemployment is due to a pre-existing disability or limitation rather than long COVID. Regardless of the cause, the findings of the present study underscore the importance of future exploration into the causes and mitigators for functional limitations with long COVID given the potential for negative economic impact for affected individuals.

In this study, people covered by public health insurance had higher odds than uninsured individuals of functional impairment (OR = 5.3 [95% CI = 1.3, 22.1]), although the uninsured group was small and the confidence interval is wide, so this should be interpreted cautiously. Studies of people with long COVID have described that they are less likely to be uninsured and more likely to access healthcare than people without long COVID [22]. Moreover, this could again be a case of reverse causation because individuals with significant pre-existing physical limitations may be more likely to qualify for public programs such as Medicaid, and there were a meaningful number of individuals over 65 years of age in this cohort, so Medicare was also likely a common insurer. Likewise, pain interference also had a significant association with functional limitations in this population, with the odds increasing as pain interference worsened (little pain interference versus none, OR = 2.3 [95% CI = 1.3, 4.1]; moderate interference versus none, OR = 8.8 [95% CI = 4.5, 17.1]; and extreme/quite a bit of interference versus none, OR = 16.9 [95% CI = 8.3, 34.4]). Pain is certainly a factor that can lead to functional limitation, so it follows that this association would be significant, but this finding should be interpreted with caution given the close relationship between pain and functional limitations.

Another noteworthy finding in this analysis was the lack of any association between functional status and a variety of variables: exercise status, smoking, age, sex, race, ethnicity, education level, and physical and mental health status. Female sex is a frequently reported factor associated with functional limitations in long COVID, but it was not a significant association in this study. Moreover, other factors that could perhaps be associated with overall health and quality of life, such as exercise status or physical or mental health status, were not associated with functional limitation.

## 5. Limitations

There were several limitations in this study. First, the cross-sectional study design can demonstrate a statistical association but not causality. The use of self-reported data within MEPS is also a limitation, as it introduces the potential for self-reporting and recall bias. Furthermore, long COVID was a relatively new condition when data were collected in 2023, so there may have been a lack of understanding when discussing this variable with participants. In addition, several limitations exist around the functional limitation metric in the MEPS data. The cause of the functional limitations reported by participants is not known and may have been unrelated to long COVID. Similarly, there is no information gathered by MEPS regarding treatments for the acute COVID episode or long COVID. This may be relevant because treatment of acute COVID reduces the rates of long COVID and its sequelae and contributes to rehabilitation strategies [23,24]. For instance, one article from Bulgaria indicated that colchicine had an inverse relationship with hospitalization and post-COVID symptoms [25]. Therefore, the various possible treatments for COVID should be considered in future research. Functional limitation was only available as a dichotomous variable rather than a count variable, which could have led to a more informative analysis. Finally, functional limitation was described to MEPS participants as a physical limitation, so other forms of functional limitations that are defined by the NCHS (such as hearing and cognition) may not have been well-captured in these data.

## 6. Conclusions

In this cross-sectional analysis of MEPS data, 26% of adults with MEPS-defined long COVID reported functional limitations, although whether the limitation was due to long COVID is unknown. Factors specifically associated with functional limitations in this population included non-married status (versus married), having 2–4 chronic health conditions (versus 0–1 conditions), being unemployed (versus employed), having public health insurance (versus uninsured), and reporting pain interference (versus no pain). Further research is needed to better understand factors predictive of functional limitations in people with long COVID and to explore the scope and severity of these limitations.

## Figures and Tables

**Table 1:** Characteristics of US adults with long COVID stratified by functional limitation status.

Variable	Functional Limitation % [95% CI] *n* = 271	No Functional Limitation % [95% CI] *n* = 773	*p*
Age			<0.0001
70+	21.1 [15.5, 26.7]	9.5 [7.1, 11.9]	
60–69	26.3 [19.2, 33.5]	15.8 [13.0, 18.6]	
50–59	25.6 [19.1, 32.1]	20.9 [16.6, 25.1]	
40–49	10.9 [6.1, 15.8]	17.8 [14.9, 20.7]	
18–39	16.0 [8.9, 23.1]	36.0 [31.5, 40.6]	
Sex			0.8138
Male	43.5 [35.3, 51.8]	42.4 [38.0, 46.8]	
Female	56.5 [48.2, 64.7]	57.6 [53.2, 62.0]	
Race			0.0444
White	85.3 [79.7, 91.0]	82.0 [78.6, 85.4]	
Black	8.8 [4.5, 13.1]	7.2 [5.3, 9.2]	
Asian	1.2 [0.1, 2.5]	6.0 [3.5, 8.5]	
Multiple/other	4.7 [1.4, 8.0]	4.8 [2.7, 6.9]	
Ethnicity			0.0674
Hispanic	12.7 [6.6, 18.9]	19.4 [15.8, 23.1]	
Non-Hispanic	87.3 [81.1, 93.4]	80.6 [76.9, 84.2]	
Marital status			<0.0001
Never married/divorced/separated/widowed	61.0 [54.0, 68.0]	41.0 [36.5, 45.4]	
Married	39.0 [32.0, 46.0]	59.0 [54.6, 63.5]	
Education			0.0504
Up to and including high school	48.2 [39.9, 56.5]	39.0 [34.5, 43.5]	
Beyond high school	51.8 [43.5, 60.1]	61.0 [56.5, 65.5]	
Number of conditions			<0.0001
5+	27.2 [20.9, 33.4]	10.6 [8.2, 13.0]	
2–4	59.1 [51.6, 66.5]	41.7 [37.1, 46.3]	
0–1	13.8 [7.9, 19.6]	47.7 [43.1, 52.3]	
Regular exercise			0.0022
Yes	38.6 [31.1, 46.1]	52.0 [47.8, 56.3]	
No	61.4 [53.9, 68.9]	48.0 [43.7, 52.2]	
Smoking status			0.2064
Smoker	14.1 [8.6, 19.6]	10.5 [7.4, 13.7]	
Nonsmoker	85.9 [80.4, 91.4]	89.5 [86.3, 92.6]	
Employment status			<0.0001
Unemployed	64.2 [55.4, 73.0]	26.6 [22.3, 30.9]	
Employed	35.8 [27.0, 44.6]	73.4 [69.1, 77.7]	
Income			<0.0001
Poor/low	41.3 [33.1, 49.5]	23.7 [19.7, 27.7]	
Middle/high	58.7 [50.5, 66.9]	76.3 [72.3, 80.3]	
Health insurance			<0.0001
Private	52.3 [44.8, 59.9]	71.1 [67.1, 75.1]	
Public	46.6 [39.0, 54.2]	21.7 [17.9, 25.4]	
Uninsured	1.0 [0.1, 2.3]	7.2 [4.9, 9.6]	
Pain			<0.0001
Extreme/quite a bit	42.4 [34.6, 50.2]	5.8 [3.8, 7.8]	
Moderate	23.1 [15.7, 30.5]	9.0 [6.8, 11.2]	
Little	24.5 [18.0, 30.9]	29.3 [24.7, 33.8]	
None	10.1 [5.5, 14.6]	55.9 [51.3, 60.6]	
Physical health status			<0.0001
Fair/poor	43.4 [35.1, 51.7]	15.0 [11.7, 18.3]	
Good	41.1 [33.8, 48.5]	38.0 [34.2, 41.8]	
Very good	13.4 [8.8, 18.0]	33.6 [29.5, 37.7]	
Excellent	2.1 [0.1, 4.9]	13.4 [10.1, 16.7]	
Mental health status			<0.0001
Fair/poor	28.3 [21.7, 34.9]	13.6 [10.7, 16.5]	
Good	35.7 [27.9, 43.5]	33.0 [28.9, 37.1]	
Very good	25.0 [19.0, 31.0]	35.2 [30.6, 39.7]	
Excellent	11.0 [6.0, 15.9]	18.2 [14.5, 22.0]	

**Table 2:** Associations of each variable with having a functional limitation versus not having a functional limitation among US adults with long COVID.

Variable	OR [95% CI]
Age: 70+ vs. 18–39	1.3 [0.5, 3.1]
Age: 60–69 vs. 18–39	1.1 [0.5, 2.5]
Age: 50–59 vs. 18–39	1.1 [0.5, 2.1]
Age: 40–49 vs. 18–39	1.5 [0.6, 3.7]
Sex: male vs. female	1.4 [0.8, 2.2]
Race: White vs. multiple/other	1.6 [0.6, 4.5]
Race: Black vs. multiple/other	1.4 [0.5, 4.4]
Race: Asian vs. multiple/other	0.7 [0.1, 4.0]
Ethnicity: Hispanic vs. non-Hispanic	0.8 [0.4, 1.8]
Marital status: never married/divorced/separated/widowed vs. married	**1.8 [1.2, 2.8]**
Education: up to and including high school vs. beyond high school	1.1 [0.6, 1.8]
Number of conditions: 5+ vs. 0–1	1.6 [0.7, 3.5]
Number of conditions: 2–4 vs. 0–1	**2.8 [1.4, 5.6]**
Regular exercise: yes vs. no	0.9 [0.6, 1.5]
Smoking status: smoker vs. nonsmoker	0.9 [0.4, 1.8]
Employment status: unemployed vs. employed	**3.5 [2.0, 6.4]**
Income: poor/low vs. middle/high	0.8 [0.4, 1.4]
Health insurance: private vs. uninsured	3.9 [0.9, 16.4]
Health insurance: public vs. uninsured	**5.3 [1.3, 22.1]**
Pain interference: extreme/quite a bit vs. none	**16.9 [8.3, 34.4]**
Pain interference: moderate vs. none	**8.8 [4.5, 17.1]**
Pain interference: little vs. none	**2.3 [1.3, 4.1]**
Physical health: fair/poor vs. excellent	4.2 [0.7, 24.2]
Physical health: good vs. excellent	2.8 [0.6, 13.7]
Physical health: very good vs. excellent	1.2 [0.2, 6.4]
Mental health: fair/poor vs. excellent	0.6 [0.3, 1.4]
Mental health: good vs. excellent	0.9 [0.4, 1.8]
Mental health: very good vs. excellent	0.9 [0.5, 1.9]

Wald statistic: *p* < 0.0001. c statistic: 0.872. OR = odds ratio. CI = confidence interval. Bold font indicates statistical significance.

## Data Availability

The raw data supporting the conclusions of this article will be made available by the authors upon request. These data were derived from the following resource available in the public domain: Medical Expenditure Panel Survey (MEPS) HC-251 2023 Full-Year Consolidated Data File at https://meps.ahrq.gov/mepsweb/data_stats/download_data_files_detail.jsp?cboPufNumber=HC-25 (accessed on 28 August 2026).

## Abbreviations

The following abbreviations are used in this manuscript:

MEPS	Medical Expenditure Panel Survey
NCHS	National Center for Health Statistics
US	United States

## References

[1] National Academies of Sciences, Engineering, and Medicine (NASEM) (2024). A Long COVID Definition: A Chronic, Systemic Disease State with Profound Consequences.

[2] National Center for Health Statistics (NCHS) Functional Limitations. https://www.cdc.gov/nchs/hus/topics/functional-limitation.htm.

[3] Backe I.F., Patil G.G., Nes R.B., Clench-Aas J. (2017). The relationship between physical functional limitations, and psychological distress: Considering a possible mediating role of pain, social support and sense of mastery. SSM Popul. Health.

[4] Edemekong P.F., Bomgaars D.L., Sukumaran S., Schoo C. (2025). Activities of Daily Living. StatPearls.

[5] Ramnath U., Rauch L., Lambert E.V., Kolbe-Alexander T.L. (2018). The relationship between functional status, physical fitness and cognitive performance in physically active older adults: A pilot study. PLoS ONE.

[6] Eekhoff E.M.W., van Schoor N.M., Biedermann J.S., Oosterwerff M.M., de Jongh R., Bravenboer N., van Poppel M.N.M., Deeg D.J.H. (2019). Relative importance of four functional measures as predictors of 15-year mortality in the older Dutch population. BMC Geriatr..

[7] Banić M., Janković Makek M., Samaržija M., Muršić D., Boras Z., Trkeš V., Baričević D., Koršić M., Basara L., Jalušić T. (2022). Risk factors and severity of functional impairment in long COVID: A single-center experience in Croatia. Croat. Med. J..

[8] Ford N.D., Slaughter D., Edwards D., Dalton A., Perrine C., Vahratian A., Saydah S. (2023). Long COVID and Significant Activity Limitation Among Adults, by Age—United States, June 1–13, 2022, to June 7–19, 2023. MMWR Morb. Mortal. Wkly. Rep..

[9] Beauchamp M., Kirkwood R., Duong M., Ho T., Raina P., Kruisselbrink R., Jones A., Girolametto C., Costa A. (2024). Long-Term Functional Limitations and Predictors of Recovery After COVID-19: A Multicenter Prospective Cohort Study. Am. J. Med..

[10] Prazeres F., Romualdo A.P., Campos Pinto I., Silva J., Oliveira A.M. (2025). The impact of long COVID on quality of life and work performance among healthcare workers in Portugal. PeerJ.

[11] Ortega-Martin E., Alvarez-Galvez J. (2026). Understanding quality-of-life patterns in long COVID: How Symptoms and socioeconomic conditions shape patient wellbeing. PLoS ONE.

[12] Vélez-Santamaría R., Fernández-Solana J., Méndez-López F., Domínguez-García M., González-Bernal J.J., Magallón-Botaya R., Oliván-Blázquez B., González-Santos J., Santamaría-Peláez M. (2023). Functionality, physical activity, fatigue and quality of life in patients with acute COVID-19 and Long COVID infection. Sci. Rep..

[13] Laskovski L., Felcar J.M., Fillis M.M.A., Trelha C.S. (2023). Risk factors associated with limited functional status among out-of-hospital patients 30 days and one year after a diagnosis of COVID-19: A cohort study. Sci. Rep..

[14] Mazer B., Feldman D.E. (2025). Demographic and Clinical Factors Associated with Functional Changes in Long-COVID. Am. J. Phys. Med. Rehabil..

[15] Xiang J., Zheng H., Cai Y., Chen S., Wang Y., Chen R. (2025). Cumulative social disadvantage and its impact on long COVID: Insights from a U.S. national survey. BMC Med..

[16] Agency for Healthcare Research and Quality Medical Expenditure Panel Survey (MEPS) Survey Background. https://meps.ahrq.gov/data_stats/publications.jsp.

[17] Agency for Healthcare Research and Quality Medical Expenditure Panel Survey (MEPS) HC-251 2023 Full Year Consolidated Data File. https://meps.ahrq.gov/mepsweb/data_stats/download_data_files_detail.jsp?cboPufNumber=HC-251.

[18] Agency for Healthcare Research and Quality Medical Expenditure Panel Survey (MEPS) HC-251 2023 Full Year Consolidated Data Codebook. https://meps.ahrq.gov/mepsweb/data_stats/download_data/pufs/h251/h251cb.pdf.

[19] Kudoh R., Komiya K., Shinohara A., Kageyama T., Hiramatsu K., Kadota J.I. (2023). Marital status and post-COVID-19 conditions. Respir. Investig..

[20] Jace C.E., Makridis C.A. (2021). Does marriage protect mental health? Evidence from the COVID-19 pandemic. Soc. Sci. Q..

[21] Perlis R.H., Lunz Trujillo K., Safarpour A., Santillana M., Ognyanova K., Druckman J., Lazer D. (2023). Association of Post-COVID-19 Condition Symptoms and Employment Status. JAMA Netw. Open.

[22] Ford N.D., Slaughter D., Dalton A.F., Edwards D., Ma K., King H., Saydah S. (2024). Health Insurance and Access to Care in U.S. Working-Age Adults Experiencing Long COVID. Am. J. Prev. Med..

[23] Bramante C.T., Buse J.B., Liebovitz D.M., Nicklas J.M., Puskarich M.A., Cohen K., Belani H.K., Anderson B.J., Huling J.D., Tignanelli C.J. (2023). Outpatient treatment of COVID-19 and incidence of post-COVID-19 condition over 10 months (COVID-OUT): A multicentre, randomised, quadruple-blind, parallel-group, phase 3 trial. Lancet Infect. Dis..

[24] Chakraverty S., Chynkiamis N., Maru N., Megaritis D., Hume E., Cucato G., Wilhelm E.N., Vogiatzis I. (2026). Rehabilitation in long COVID: A systematic review and meta-analysis. ERJ Open Res..

[25] Mitev V., Marinov K., Tiholov R., Tashkov K., Bilyukov R., Lilov A.I., Palaveev K.R., Miteva A., Miteva I., Dimitrova V.S. (2025). High colchicine doses are more effective in COVID-19 outpatients than nirmatrelvir/ritonavir, remdesivir, and molnupiravir. Pharmacia.

